# Factors associated with disease evolution in Greek patients with inflammatory bowel disease

**DOI:** 10.1186/1471-230X-6-21

**Published:** 2006-07-25

**Authors:** Constantinos Chatzicostas, Maria Roussomoustakaki, Spiros Potamianos, Gregorios Paspatis, Ioannis Mouzas, John Romanos, Helen Mavrogeni, Elias Kouroumalis

**Affiliations:** 1 Department of Gastroenterology, General Hospital of Rethymnon, Rethymnon, Crete, Greece; 2 Department of Gastroenterology, University Hospital of Heraklion, Heraklion, Crete, Greece; 3 Department of Gastroenterology, Venizelion General Hospital, Heraklion, Crete, Greece; 4 Department of Surgical Oncology, University Hospital of Heraklion, Heraklion, Crete, Greece; 5 Department of Internal Medicine, General Hospital of Rethymnon, Crete, Greece

## Abstract

**Background:**

The majority of Crohn's disease patients with B1 phenotype at diagnosis (i.e. non-stricturing non-penetrating disease) will develop over time a stricturing or a penetrating pattern. Conflicting data exist on the rate of proximal disease extension in ulcerative colitis patients with proctitis or left-sided colitis at diagnosis. We aimed to study disease evolution in Crohn's disease B1 patients and ulcerative colitis patients with proctitis and left-sided colitis at diagnosis.

**Methods:**

116 Crohn's disease and 256 ulcerative colitis patients were followed-up for at least 5 years after diagnosis. Crohn's disease patients were classified according to the Vienna criteria. Data were analysed actuarially.

**Results:**

B1 phenotype accounted for 68.9% of Crohn's disease patients at diagnosis. The cumulative probability of change in disease behaviour in B1 patients was 43.6% at 10 years after diagnosis. Active smoking (Hazard Ratio: 3.01) and non-colonic disease (non-L2) (Hazard Ratio: 3.01) were associated with behavioural change in B1 patients. Proctitis and left-sided colitis accounted for 24.2%, and 48.4% of ulcerative colitis patients at diagnosis. The 10 year cumulative probability of proximal disease extension in patients with proctitis and left-sided colitis was 36.8%, and 17.1%, respectively (p: 0.003). Among proctitis patients, proximal extension was more common in non-smokers (Hazard Ratio: 4.39).

**Conclusion:**

Classification of Crohn's disease patients in B1 phenotype should be considered as temporary. Smoking and non-colonic disease are risk factors for behavioural change in B1 Crohn's disease patients. Proximal extension is more common in ulcerative colitis patients with proctitis than in those with left-sided colitis. Among proctitis patients, proximal extension is more common in non-smokers.

## Background

Inflammatory bowel diseases (IBD) are chronic heterogeneous disorders with unpredictable clinical course. Studies on Crohn's disease (CD) behaviour have been hampered by the variability of classifications used and by their unsatisfactory degree of inter-rater agreement [1-4]. The Vienna classification of CD [5] has been proposed in an effort to stratify patients on the basis of widely accepted and reproducible criteria [6]. Although CD location, as defined by the Vienna classification is a relatively stable phenotype, its behaviour varies over time [7]. The majority of patients with B1 phenotype (i.e. non-stricturing non-penetrating disease) will develop over time a stricturing or a penetrating pattern (i.e. B2 and B3 respectively) [7-11]. The environmental and genetic factors affecting disease behaviour evolution are not fully characterized. Ileal location (i.e. L1) [8,9,11], active smoking [8] and number of flares per year [8] were reported to be the major determinants of phenotypic changes. Moreover, some authors suggested that intra-abdominal penetrating disease (IAPD) and perianal penetrating disease (PAPD) represent distinct clinic entities which should be studied independently [8,11].

The extent of colonic involvement in ulcerative colitis (UC) patients is clinically relevant because it has some bearing on the severity of disease and on the needs for medical supervision, drug therapy and surgical excision [12-14]. Although in certain patients with proctitis and left-sided UC, the inflammation spreads proximally, studies on the stability of disease extent over time and risk factors influencing disease extent progression have failed to offer conclusive information [12-21].

We aimed to study the long-term outcome of a homogeneous and well-defined group of Cretan patients with IBD. Particular emphasis was given on the study of rate and risk factors that might influence: change of disease behaviour over time in B1 CD patients; and proximal disease extension in UC patients with proctitis and left-sided colitis at diagnosis;

## Methods

The Gastroenterology Department of the University Hospital of Heraklion has previously conducted prospective and population-based epidemiologic studies of UC and CD in the prefecture of Heraklion, and participated in the European collaborative study of IBD (EC-IBD) [22-26].

The patient cohort seen at the aforementioned institution has been enriched with cases followed-up at the Gastroenterology Department of Venizelion General Hospital of Heraklion (since its opening at 1996). During the study period these two units were the sole centers providing health care for patients with GI tract problems living in the island of Crete (approximately 600 000 people). Clinical details were collected in a special designed form, by patient interview and case note review. Our study protocol was approved by the Ethics Committee of the Medical School of the University of Crete. Written consent was obtained from the patients.

Of the 736 patients listed in the IBD registry (498 with UC and 238 with CD), 372 (256 with UC and 116 with CD) met the following criteria for inclusion in this analysis:

1. A confirmed diagnosis of UC or CD by standard clinical, radiologic, endoscopic and histologic criteria. Patients with an initial diagnosis of UC later changed to CD or vice versa were included in the analysis with the final diagnosis.

2. A satisfactory assessment of the extent of disease at the initial examination in patients with UC and of disease behaviour at diagnosis in patients with CD. Extent, location, and behaviour of UC and CD were evaluated by ileo-colonoscopy, upper GI tract endoscopy, enteroclysis, CT scan of abdomen and surgical reports.

3. A follow-up period of at least 60 months. Time of diagnosis was defined as the date of first detection of unequivocal inflammatory abnormalities of the intestine, as assessed from radiologic, endoscopic, or operative observations [9].

The extent of UC was defined as proctitis (rectum involvement), left-sided colitis (involvement up to the splenic flexure) and extensive colitis (involvement proximal to the splenic flexure). Proximal disease extension was defined as the finding at any follow-up colonoscopy of macroscopic inflammation extending beyond 15 cm from the anus in case of proctitis or beyond the splenic flexure in case of left-sided colitis.

The clinical classification of patients with CD was carried out in strict accordance with the Vienna criteria [5]. In patients with B1 phenotype at diagnosis the first morphologic demonstration of narrowing or penetrating complication was used to mark the occurrence of the complication. Some patients had stricturing disease and then developed a fistula. The sequence was taken into account (i.e. B2 then B3 disease) when the time interval between the two events was greater than one year, otherwise the disease was considered as B3 [9]. According to previous analyses, patients who were diagnosed with PAPD before CD diagnosis were assumed to have developed their fistula on the day after diagnosis [27]. Only major operations (i.e. bowel resections) were considered as surgical therapy in patients with CD.

Patients were seen regularly depending on their needs, and had easy access in flare-up episodes. At each visit, symptoms and signs, laboratory and diagnostic examinations, medications and surgery were prospectively registered on a predesigned data sheet. Asymptomatic patients were fully investigated on a yearly basis. Finally, an assessment was made for each year of follow-up, comprising the clinical activity within the year, disease course, intestinal complications, extent of disease, diagnostic procedures, medical treatments, operations, hospitalizations, and outpatient visits.

Chronic continuous course was defined as continuous activity within and during each year of follow-up [28]. Patients were defined as smokers if they consumed at least 7 cigarettes/week and non-smokers if they had never smoked or had stopped smoking before diagnosis [8]. A familial form of the disease was defined as the presence of UC or CD in a relative, whatever the familial degree (i.e. parent, child, sibling, grandparent, grandchild, aunt, uncle, and cousin).

## Statistical analysis

Descriptive statistics are expressed as mean and standard deviation or as frequency counts and percentages. Categorical data were analyzed using Pearson's chi-square test. Equalities of means and proportions were tested using Student's t test and analysis of variance with Bonferroni correction for group comparisons. P values of less than 0.05 were considered statistically significant.

Survival analyses, using the Kaplan-Meier method with log-rank test [29,30], and Cox's proportional hazards model for multivariate analyses [31] were used to study the predefined end-points. Patients with UC were included in these calculations only as long as they had not undergone colectomy. Patients not reaching the end point under consideration were censored at the time of the last clinical follow-up. The multivariate analysis was applied to those of the variables having a p = 0.2 in order to identify possible confounding effects [32]. In the multivariate procedure, p = 0.05 was considered as the level of significance. Results of analysis are presented as hazard ratios (HR) with 95% confidence intervals (95% CI). The following variables suspected as possible predictors were included in the analysis: age at diagnosis, months from onset to diagnosis, sex, positive family history of IBD, active smoking, disease location in patients with CD, need for immunosuppressive therapy, chronic continuous course, and previous major operation in patients with CD. To examine an age-related effect, patients with CD were classified as the A1/A2 Vienna classification of under or over 40 years of age. Continuous variables (i.e. age at diagnosis in patients with UC and months from onset to diagnosis in patients with UC and CD) were dichotomized by splitting mean values, which were then compared.

## Results

### Crohn's disease patients

**Figure 1:**
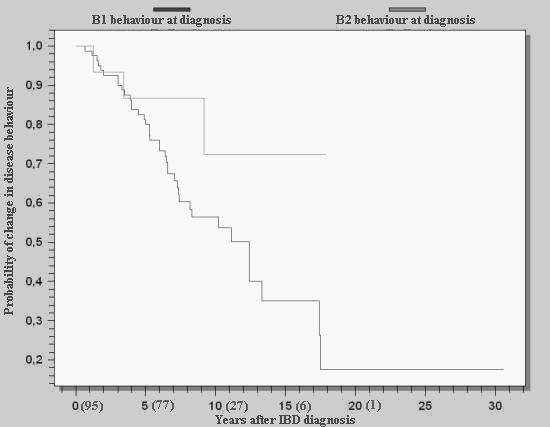
Kaplan-Meier estimates of remaining free of change of disease behaviour in patients with B1 and B2 phenotypes at diagnosis. The numbers in parentheses (after years from IBD diagnosis) refer to the numbers of patients at risk at the corresponding time points. Log-rank test p value: 0.073.

A total of 116 patients with CD were included in the study (Table 1). B1 phenotype accounted for 68.9% of CD patients at diagnosis (Table 1), for 55.1% of cases at 5 years after diagnosis (i.e. 64 patients), and for 36.8% of cases at the end of the study (i.e. 42 patients). Eleven patients with B1 behaviour at diagnosis (13.7%) developed B2 disease, whereas 27 patients (33.7%) developed B3 disease. Survival analysis showed that 10 years after diagnosis, the proportion of CD patients still remaining B1 was 38.9% (± Standard Error (SE): 4.9%). The cumulative probability of disease behaviour reclassification in patients with B1 phenotype at diagnosis was 43.6% (± SE: 6.1%) at 10 years after diagnosis (Figure 1).

Factors associated with phenotype change in patients with B1 disease at diagnosis as identified in univariate analysis are listed in table 2. Regression model analysis selected active smoking (HR: 3.01; 95% CI: 1.40–6.45; P: 0.005) and non-L2 disease (HR: 3.01; 95% CI: 1.16–7.78; P: 0.023) as significantly associated with B2 and B3 reclassification in patients with B1 phenotype at diagnosis. Active smoking (HR: 3.84; 95% CI: 1.00–14.72; P: 0.049) and L1 disease (HR: 5.76; 95% CI: 1.65–20.11; P: 0.006) were selected as significantly associated with reclassification from B1 to B2 phenotype, whereas, active smoking (HR: 3.68; 95% CI: 1.45–9.29; P: 0.006) and non-L2 disease (HR: 3.74; 95% CI: 1.11–12.56; P: 0.033) were selected as significantly associated with reclassification from B1 to B3 phenotype.

Fifteen patients with initially B1 phenotype (18.7%) developed IAPD and 12 patients (15%) PAPD. Regression model analysis selected active smoking (HR: 5.18; 95% CI: 1.12–23.96; P: 0.035), L1 disease (HR: 5.87; 95% CI: 1.41–24.42; P: 0.015) and chronic continuous course (HR: 4.56; 95% CI: 1.25–16.61; P: 0.021) as significantly associated with the development of IAPD in patients with initially B1 phenotype. In univariate analysis the only factor significantly associated with reclassification from B1 to PAPD was active smoking (P: 0.049), which was however rejected from the regression model (HR: 3.21; 95% CI: 0.96–10.71; P: 0.058) when it was combined with other variables having a p value between 0.51–0.2.

None of our B1 patients reclassified as B2 became B3, whereas 3 out of 15 patients with B2 phenotype at diagnosis became B3. The cumulative probability of B3 reclassification in patients with B2 phenotype at diagnosis was 27.8% (± SE: 15.1%) at 10 years after diagnosis (Figure 1).

At the end of follow-up, 51 patients had B3 phenotype. Overall, B3 phenotype remained unchanged in 23 patients with IAPD and 17 patients with PAPD. However, 5 out of 28 patients (17.8%) with IAPD developed subsequently PAPD, and 6 out of 23 patients (26%) with PAPD developed subsequently IAPD. The cumulative probability of appearance of the other penetrating phenotype in an already B3 patient was 22.7% (± SE: 6.7%) and 32.4% (± SE: 10.8%) at 5 and 10 years, respectively. The probability of appearance of PAPD in patients with IAPD was not related to age at diagnosis, disease location, or active smoking. Similarly, the probability of appearance of IAPD in patients with PAPD was not related to age at diagnosis or active smoking. Although this was also the case for patients with L1, L2 and L4 disease, L3 patients with PAPD had a greater risk of developing IAPD (P: 0.021).

**Figure 2:**
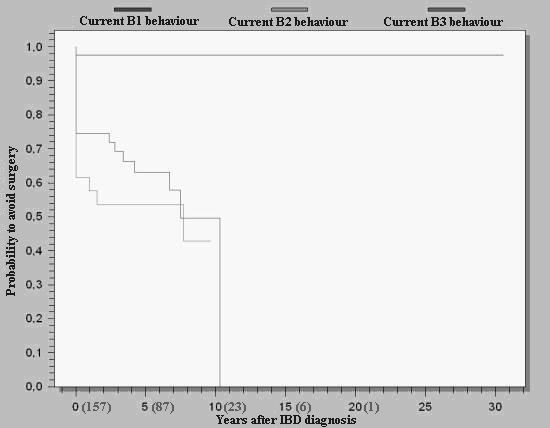
Kaplan-Meier estimates of remaining free of surgery with regard to current disease behaviour. In patients with B1 disease (upper curve), years refer to years after diagnosis in those with stable behaviour, and to years until change of behaviour in those reclassified; In patients with B2 disease (lower curve), years refer to years after diagnosis in cases with stable B2 phenotype, and to years after change of disease behaviour from B1 to B2 in those reclassified; In patients with B3 disease (middle curve), years refer to years after diagnosis in cases with initial B3 phenotype, and to years after change of disease behaviour form B1 or B2 to B3 in those reclassified; The numbers in parentheses (after years from IBD diagnosis) refer to the numbers of patients at risk at the corresponding time points. The corresponding log-rank test p values are: <0.001 (B1 vs B2), <0.001 (B1 vs B3), 0.359 (B2 vs B3).

At 5 years of follow-up, 23 out of 116 CD patients (19.8%) had experienced a major surgery. The cumulative probability of surgery was 29.7% (± SE: 4.6%) at 10 years after diagnosis. Patients with L2 disease had a lower risk of surgery than patients with L1 (P: < 0.001), L3 (P: 0.011), and L4 disease (P: 0.026). The risk of surgery was also higher in current smokers (P: < 0.001). As shown in figure 2, the risk of surgery was significantly higher in patients with current B2 and B3 phenotypes when compared to that of patients with current B1 phenotype, whereas the difference between B2 and B3 curves was insignificant.

Six patients underwent a second operation. The cumulative probability of 2^nd ^intestinal resection was 7.4% (± SE: 5%) and 17.2% (± SE: 7.9%) at 5 and 10 years after 1^st ^intestinal resection, respectively.

### Ulcerative colitis patients

**Figure 3:**
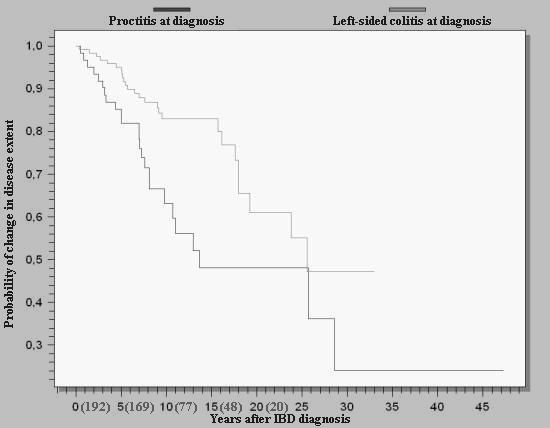
Kaplan-Meier estimates of remaining free of proximal disease extension in patients with proctitis and left-sided colitis at diagnosis. The numbers in parentheses (after years from IBD diagnosis) refer to the numbers of patients at risk at the corresponding time points. Log-rank test p value: 0.003.

A total of 256 patients with UC were included in the study (Table 3). During follow-up, proximal disease extension was documented in 25 out of 62 patients (40.3%) with proctitis and in 26 out of 124 patients (20.9%) with left-sided colitis. At 5 years of follow-up, proximal extension was documented in 11 out of 62 patients with proctitis (17.7%) and in 7 out of 124 patients with left-sided colitis (5.6%). The cumulative probability of proximal extension in patients with proctitis was 36.8% (± SE: 7.1%) at 10 years after diagnosis (Figure 3). In contrast, the cumulative probability of proximal extension in patients with left-sided colitis was significantly lower, 17.1% (± SE: 3.8%) at 10 years after diagnosis (Figure 3). None of our proctitis patients eventually underwent colectomy. In contrast, one patient with left-sided colitis at diagnosis and proximal disease extension during follow-up, and two other left-sided colitis patients without further spread of the disease within the observation period were operated on. The 10 year cumulative probability of surgery in our ulcerative colitis patient cohort was 2.9% (± SE: 1.7%) (years refer to time interval after the initial 5 year period of minimum follow-up from diagnosis), whereas, the cumulative probability of colorectal cancer was 4.5% (± SE: 3.6%) at 20 years

Factors associated with disease progression in proctitis patients as identified in univariate analysis are listed in table 4. Regression model analysis selected non-smoking as significantly associated with disease progression in patients with proctitis (HR: 4.39; 95% CI: 1.02–18.79; P: 0.046). None of the variables under study was significantly associated with disease progression in patients with left-sided colitis.

## Discussion

Many patients with severe GI problems from other parts of mainland Greece seek medical care in Athens, thus by strict criteria the patients included in series originating from referral Athenian hospitals cannot be considered to be a representative sample for all cases with IBD in Greece [33-37]. It is a well known fact that patients with benign clinical course are under-represented at a tertiary referral center [38]. The advantage of the present study is that, our population is dispersed over a small geographical area and referred to only two Gastroenterology Units which have registered practically all patients with IBD in Crete.

### Crohn's disease

There is only one study analyzing the natural history of CD in Greece [33]. In this series, only crude rates were reported, and emphasis was given on disease location, not behaviour.

Progression over time of CD pathologic changes has been suggested in a previous report by Kelly et al [39]. Other series, using the Vienna [7,9,11] or other classifications [27,40] have also focused on the changing nature of CD over time. Although our results fit well with those previously reported, they should be interpreted with caution as only 22 patients with B1 behaviour and 5 patients with B2 behaviour were available for follow-up at 10 years after diagnosis. Some authors found that patients who are classified as B2 at diagnosis tend to remain B2 over time [8,41]. Although these observations are suggestive more of a parallel than of a sequential progression from B1 to either B2 or B3 behaviours, it is quite clear that at least some patients with stricturing behaviour at presentation will finally develop penetrating disease [8,41,42].

An important factor for determining disease behaviour is the location of lesions. In our series, active smoking and L1 or non-L2 disease were associated with change of disease behaviour in patients with B1 phenotype at diagnosis. Location and smoking have also been reported by others as factors associated with complicated CD [8,9,11,40,43,44]. Tobacco use has been a risk factor for ileal rather than colonic-only disease [45-47].

The cumulative probability of surgery in our CD patients was lower than that reported in other series [48-51]. Once again, our results should be interpreted with caution as only 43 patients were available for follow-up at 10 years after diagnosis. As expected, the probability of surgery was significantly higher in patients with either B2 or B3 phenotypes (Figure 2). The probability of surgery in B1 patients without change of disease behaviour was minimal, a finding quite similar to that previously reported by others [11,49]. Smoking has been previously shown to be associated with increased risk of surgery in CD patients [40,45,47,52,53]. The lower probability of surgery in patients with colonic disease has been previously reported by other authors using different classifications [40,48,50,54,55].

The risk for a second resection in our patients previously operated on for CD was lower than that reported in other series [53,56]. Previous reports using different classifications identified more frequent reoperation in those with perforating disease [1,57,58], but others have failed to confirm this association [59,60].

Both intra-abdominal and perianal fistulas were classified by the Vienna Working Party Group as penetrating disease [5]. However, some studies suggested that they represent distinct entities [8,11,61]. We found that in a significant proportion of B3 patients both penetrating phenotypes appear. Although this was also the case in other reports, in some series no data were clearly reported [27,62], whereas in other series only crude rates were reported [8,9,11,49,63].

### Ulcerative colitis

There are four Athenian studies investigating the clinical course of UC [34-37]. In all these series only crude rates were reported. The age and distribution of macroscopic inflammation at presentation in our patient cohort fits well with that reported in other series [25,26]. The rate of colectomy seems quite low in our patient cohort, especially when compared to other much larger series [12]. However, the rate of colectomy after 5 years is well known to be quite small, about 1% per year [64,65], and this low rate of colectomy equally applies to patients with initial distal disease and those with initial pancolitis [64]. The cumulative probability of colorectal cancer in our patients falls within the wide confidence intervals calculated by others [65,66].

Conflicting data exist on the rate of proximal disease extension of ulcerative proctitis. [13-16,20,21]. When considering only those two studies in which diagnosis and disease progression were confirmed using endoscopy and survival analysis was carried out, the cumulative rates of proximal extension was reported to range between 30 and 54% at 10 years, and between 50% and 84% at 20 years after diagnosis [20,21]. Our results fit well with those previously reported, however only 19 patients with proctitis at diagnosis were available for follow-up at 10 years after diagnosis. We found that the risk of progression was higher in non-smokers, a finding previously confirmed by some [18,20] but not all authors [13]. There are several possible explanations for these conflicting results. Some are related to differences in disease extent definition (true proctitis [15,18-21] vs proctosigmoiditis [12-14]) and means of follow-up surveillance (endoscopic [17,19-21] vs radiologic [18] vs a combination of sigmodoscopy and barium enema [12-15]). Furthermore, there is poor agreement between extent assessed by histology and endoscopy both at diagnosis and at follow-up [17,19,25].

Fifty eight of our ulcerative colitis patients were available for follow-up at 10 years after diagnosis. Thus, we have found the cumulative probability of proximal extension in these patients to be 17.1% at 10 years after diagnosis. Others reported cumulative rates ranging between 14% and 32% at 10 years after diagnosis [13,14]. However, these figures were evaluated using barium enemas and calculated for mixed groups of patients with proctitis and procto-sigmoiditis [13], or with left-sided and extensive colitis at diagnosis [14]. We were unable to find a single demographic or clinical factor associated with change of disease extent from left-sided to extensive colitis.

## Conclusion

Classification of Crohn's disease patients in B1 phenotype should be considered as temporary. Smoking and non-colonic disease are risk factors for behavioural change in B1 Crohn's disease patients. At least some patients with stricturing behaviour will finally develop penetrating disease. In a significant proportion of B3 patients both penetrating phenotypes appear. Proximal extension is more common in ulcerative colitis patients with proctitis than in those with left-sided colitis. Among proctitis patients, proximal extension is more common in non-smokers. Further studies are required to identify factors associated with proximal disease extension in ulcerative colitis patients with left-sided colitis at diagnosis.

## Abbreviations

IBD, inflammatory bowel diseases; CD, Crohn's disease; UC, ulcerative colitis; IAPD, intra-abdominal penetrating disease; PAPD, perianal penetrating disease; HR, hazard ratios

## Competing interests

The author(s) declare that they have no competing interests.

## Authors' contributions

CC: carried out the collection, statistical analysis and interpretation of data and drafting the manuscript

MR: has made substantial contributions to conception and design and revising the manuscript

SP: carried out the collection of data, the endoscopic and clinical diagnosis of the first IBD patients

GP: has been involved in the collection of data from Venizelion Hospital

IM: has been involved in acquisition of data

JR: carried out the majority of surgical procedures in all IBD patients

HM: has been involved in the collection of data from the area of Rethymnon

EK: the supervisor, has given final approval of the version to be published

## Pre-publication history

The pre-publication history for this paper can be accessed here:



## Figures and Tables

**Table 1:** Demographic and clinical parameters depending on Crohn's disease behaviour at diagnosis

	**Disease behaviour at diagnosis**			
**Variable**	**B1 (N = 80)**	**B2 (N = 15)**	**B3 (N = 21)**	**Overall (N = 116)**

Follow-up (yr)	10.8 ± 6.5	9.9 ± 4	10.4 ± 6.9	10.5 ± 6.4
Males	46 (57.5%)	12 (80%)	12 (57.1%)	70 (60.3%)
Months to diagnosis	44.6 ± 42.4	38.8 ± 45.9	26.2 ± 36.1	40.6 ± 42.1
A1	60 (75%)	9 (60%)	14 (66.6%)	83 (71.5%)
Positive family history	10 (12.5%)	2 (13.3%)	3 (14.2%)	17 (14.6%)
Location of disease				
L1	12 (15%)	7 (46.6%)*	6 (28.5%)	25 (21.5%)
L2	25 (31.2%)	2 (13.3%)	3 (14.2%)	30 (25.8%)
L3	34 (42.5%)	5 (33.3%)	12 (57.1%)	51 (43.9%)
L4	9 (11.2%)	1 (6.6%)	0	10 (8.6%)
Current smoking	41 (51.2%)	6 (40%)	12 (57.1%)	59 (50.8%)
Continuous course	17 (21.2%)	3 (20%)	3 (14.2%)	23 (19.8%)
Extraintestinal disease^‡^	43 (53.7%)	6 (40%)	8 (38%)	57 (49.1%)
Immunosuppressives	38 (47.5%)	3 (20%)*	9 (42.8%)	50 (43.1%)
Major operations^†^	14 (17.5%)	11 (73.3%)*	10 (47.6%)*	35 (30.1%)
At diagnosis	2 (2.5%)	9 (60%)*	7 (33.3%)*	18 (15.5%)
After diagnosis	12 (15%)	2 (13.3%)	3 (14.2%)	17 (14.6%)

Values are given as mean ± SD or number of patients; Values in parentheses are percentages; N, number; A1, age at diagnosis ≤ 40 years; B1, non-stricturing non-penetrating disease; B2, stricturing disease; B3, penetrating disease; L1, ileal location; L2, colonic location; L3, ileo-colonic location; L4, disease in the upper gastro-intestinal tract; ^‡^Extraintestinal disease refers to manifestations recorded during the entire follow-up period; ^†^Major operations refer to bowel resections; *P < 0.05 vs B1;

**Table 2:** Univariate analysis of risk of change of disease behaviour in patients with B1 disease at diagnosis

	**Patients without change of disease behaviour (N = 42)**	**Patients with change of disease behaviour (N = 38)**	**P**^§^
Males	26 (61.9%)	20 (52.6%)	0.180^†^
Months to diagnosis	49.2 ± 43.5	39.6 ± 41.2	NS
A1	29 (69%)	31 (81.5%)	0.147^†^
Positive family history	4 (9.5%)	6 (15.7%)	NS
Location of disease			
L1	3 (11.9%)	9 (23.6%)	0.005
L2	20 (47.6%)	5 (13.1%)	0.006
L3	15 (35.7 %)	19 (50%)	NS
L4	4 (9.5%)	5 (13.1%)	NS
Current smoking	13 (30.9%)	28 (73.6%)	0.001
Continuous course	4 (9.5%)	13 (34.2%)	0.016
Immunosuppressives	11 (26.1%)	9 (23.6%)*	NS
Surgery at diagnosis^‡^	1 (2.3%)	1 (2.6%)	NS

Values are given as mean ± SD or number of patients; Values in parentheses are percentages; N, number; A1, age at diagnosis ≤ 40 years; L1, ileal location; L2, colonic location; L3, ileo-colonic location; L4, disease of the upper gastro-intestinal tract; *Data collected until change of disease behavior; ^§^P values from survival analysis using log-rank test; ^‡^Surgery at diagnosis refers to bowel resections; ^†^Variables having a p value between 0.51 and 0.2, although insignificant, were included in the regression analysis to avoid confounding effects;

**Table 3:** Demographic and clinical parameters depending on ulcerative colitis extent at diagnosis

**Variable**	**Proctitis (N = 62)**	**Left-sided colitis (N = 124)**	**Extensive colitis (N = 70)**	**Overall (N = 256)**
Follow-up (yr)	11.3 ± 7.4	12.5 ± 7.1	12.5 ± 8.2	12.2 ± 7.5
Males	39 (62.9%)	73 (58.8%)	42 (60%)	154 (60.1%)
Months to diagnosis	24.6 ± 40	8.9 ± 13.7*	7.7 ± 15.8*	12.4 ± 24.3
Age at diagnosis (yr)	38.3 ± 14.8	43.3 ± 15.6	40.3 ± 16.3	41.3 ± 15.7
Positive family history	8 (12.9%)	17 (13.7%)	9 (12.8%)	34 (13.2%)
Current smoking	16 (25.8%)	20 (16.1%)	15 (21.4%)	51 (19.9%)
Continuous course	4 (6.4%)	9 (7.2%)	13 (18.5%)*‡	26 (10.1%)
Extraintestinal disease^†^	11 (17.7%)	27 (21.7%)	16 (22.8%)	54 (21%)
Immunosuppressives	3 (4.8%)	12 (9.6%)	16 (22.8%)*‡	31 (12.1%)
Surgery	0	3 (2.4%)	3 (4.2%)	6 (2.3%)

Values are given as mean ± SD or number of patients; Values in parentheses are percentages; N, number; ^†^Extraintestinal disease refers to manifestations recorded during the entire follow-up period; *P < 0.05 vs proctitis; ‡ P < 0.05 vs left-sided colitis;

**Table 4:** Univariate analysis of overall risk for proximal extension in patients with proctitis at diagnosis

	**Patients with disease progression (N = 25)**	**Patients without disease progression (N = 37)**	**P **^§^
Males	15 (60%)	24 (64.8%)	NS
Urban residence	19 (76%)	21 (56.7%)	0.200^†^
Months to diagnosis	21 ± 50.4	27.1 ± 31.7	0.125^†^
Age at diagnosis (yr)	43.8 ± 15.4	34.6 ± 13.4	NS
Positive family history	2 (8%)	6 (16.2%)	NS
Current smoking	2 (8%)	14 (37.8%)	0.042
Previous appendectomy	2 (8%)	2 (5.4%)	NS
Chronic continuous course	3 (12%)	1 (2.7%)	0.189^†^
Immunosuppressive drugs	0*	1 (2.7%)	NS

Values are given as mean ± SD or number of patients; Values in parentheses are percentages; N, number; *Data collected until change of disease extent; ^§^P values from survival analysis using log-rank test; ^†^Variables having a p value between 0.51 and 0.2, although insignificant, were included in the regression analysis to avoid confounding effects;
